# Influence of Peanut Flour Enrichment and Eggs on Muffin Protein Aggregation

**DOI:** 10.3390/foods14040710

**Published:** 2025-02-19

**Authors:** Mariacinzia Rutigliano, Maria Teresa Liberatore, Flavia Dilucia, Maurizio Quinto, Sibel Kacmaz, Aldo Di Luccia, Barbara la Gatta

**Affiliations:** 1 Department of Sciences of Agriculture, Food, Natural Resources and Engineering (DAFNE), University of Foggia, Via Napoli, 25, 71122 Foggia, Italy; mariacinzia.rutigliano@unifg.it (M.R.); mariateresa.liberatore@unifg.it (M.T.L.); flavia.dilucia@unifg.it (F.D.); maurizio.quinto@unifg.it (M.Q.); adiluccia@gmail.com (A.D.L.); 2 Department of Food Engineering, Engineering Faculty, Giresun University, 28200 Giresun, Turkey; sibel.kacmaz@giresun.edu.tr

**Keywords:** protein extractability, muffins, roasted peanut flour, egg proteins, gluten network organization

## Abstract

Protein–protein interactions were investigated, evaluating the influence of eggs and peanut flour on the chemical features of muffin samples. Electrophoretic, immunochemical, and chromatographic analyses were carried out to evaluate how different proteins can interact with one another, determining changes in their extractability. In this context, two extraction buffers were employed to study the rate of protein aggregate formation. Egg coagulation properties were found to affect the rate of wheat protein solubility, while the extractability of roasted peanut flour protein was less influenced and proportional to its amount in the formulation (i.e., 4% and 20%). The findings of this investigation allowed us to assume a complex form of protein organization, characterized by a “core” of wheat and egg proteins surrounded by peanut proteins, linked together through covalent reducible bonds and hydrophobic interactions. Nevertheless, the occurrence of other types of crosslinking could not be excluded.

## 1. Introduction

Muffins have increasingly become a popular snack worldwide, consumed also as breakfast cake [1]. The main ingredients in their formulation are wheat flour, sugar, oil, and eggs [2]. Muffins can be classified as cake-like muffins, mainly prepared in commercial bakeries, and bread-like muffins, mainly home-made or prepared in small industries [3]. The difference between the two varieties lies in their different sugar and fat contents: cake-like muffins contain a higher percentage of fat and sugar than bread-like muffins, varying from 18% to 40% of fat and from 50% to 70% of sugar [4]. Sugar, fat, and other ingredients like fibers and eggs are added to the formulation to increase nutritional properties, and they can affect the mechanism of gluten network formation [5]. Furthermore, the inclusion of eggs leads to the occurrence of two phenomena, structural and allergenic. The first one is due to the content of free thiol that contributes to make the gluten network stronger [6,7]; the second one is due to the allergenic properties of egg white proteins, mainly ovomucoid (Gal d1) and ovalbumin (Gal d2) [8,9,10]. Moreover, Wouters et al. [11] reported that egg white proteins could impact the heat-induced polymerization of gluten proteins. 

The significance of investigating products incurring in more than one allergen has been further underscored by the emerging formulation comprising the mixture of different flours, such as wheat flour and peanut flour [12,13].

From a technological point of view, batter preparation and the baking process of muffins lead proteins to form supramolecular-induced protein aggregates. An additional phenomenon that must be considered for muffin preparation is the presence of two separated systems in the batter, starch and eggs, which combine to give a solid appearance to the product during baking, as a consequence of starch gelatinization and egg coagulation [14]. This architecture generated by technological processing can affect protein solubility; therefore, testing different buffers could be useful to assess this feature.

From a nutritional point of view, muffins are considered convenient and moist foods that are widely appreciated by consumers, because of their taste and soft texture [15]. In order to improve muffin formulation, several studies have been carried out to reduce the fat content or enhance the nutritional value of this product [16,17]. Moreover, the use of different vegetables to increase the fiber content and the use of flours from insects to improve the physical parameters, nutrient composition, bioactive, and sensory characteristics have also been tested [18,19,20,21].

In this study, an experiment was carried out, following the research of Rutigliano et al. [22], by preparing biscuits with the addition of peanut flour to assess how the gluten network arrangement and protein extractability could change in a dried cereal-based baked product. Therefore, a study based on electrophoretic, immunochemical, and chromatographic investigations of a complex wet cereal-based baked matrix, i.e., muffins, was carried out in this research. The aim was to obtain further information on protein–protein interactions and organization in a complex food matrix with different protein sources.

## 2. Materials and Methods

### 2.1. Muffin Preparation

Commercial soft wheat flour [protein content 8.3% (dry base)] and 12% low-fat roasted peanut flour [protein content 58% (dry base)] were used as the starting materials. Commercial caster sugar, eggs, vanilla essence, vegetable oil, and sodium bicarbonate were purchased at local supermarkets.

A traditional recipe was used to produce muffins, and the ingredients’ proportion is summarized in Table 1.

These two different percentages were used to test a low and a high content of peanut flour in terms of their compatibility with the production of a workable batter on the basis of preliminary tests.

Muffin preparation was carried out by firstly mixing the dried ingredients in a Philips Professional blender HR1565 (Philips, Amsterdam, The Netherlands) for 10 min at a slow speed (level 1), then adding baking soda, warm water, two eggs, vanilla essence, and vegetable oil, and mixing for 10 min at high speed (level 3). A heaped tablespoon (approximately 30 g) of batter was placed into an individual cupcake holder and put into an electric fan oven (Whirpool Corporation, Benton Harbor, MI, USA) at 180 °C and baked for twenty min, rotating the muffins halfway through baking. The muffins were cooled down at ambient temperature and packed in labeled food-grade bags until the analyses.

### 2.2. Protein Extraction

After a laboratory oven-dry step (70 °C) until reaching a constant weight, the muffins’ protein content was obtained by the Kjeldhal method using a conversion factor of 5.7. The analyses were carried out by an automatic digestion unit (60 min at 420 °C) and through an automatic distillation and titration system (UDK159, VELP Scientifica Srl, Usmate, Monza-Brianza, Italy). The analysis was performed in duplicate (Supplementary Table S1). For the electrophoretic analysis, samples were finely ground, obtaining a powdered sample, and the proteins were extracted in duplicate using 1 g of sample (i.e., flours or muffins) in 10 mL of buffer, according to the procedure described by Rutigliano et al. [22]. The protein content of three independent flour and muffin samples was determined using the 2D Quant-Kit™ from GE-Healthcare (GE-Healthcare Bio Sciences, Little Chalfont, UK), according to the supplier’s instructions.

### 2.3. Sodium Dodecyl Sulfate Polyacrylamide Gel Electrophoresis (SDS-PAGE) and Western Blot

SDS-PAGE and immunoblotting analyses were performed for the flour, whole egg, and muffin samples, according to the procedures described by Rutigliano et al. [22]. Electrophoretic separation was carried out for 40 min at 200 V, loading the samples onto 12% NuPAGE Bis-tris gel using MES-SDS (pH 7.3) as the running buffer (Thermo Fisher Scientific, Waltham, MA, USA). Mark 12™ and Precision Plus Protein™ Standard (Bio-Rad, Richmond, CA, USA) were used as molecular weight markers. A Typhoon Trio scanner (GE Healthcare, Buckinghamshire, UK) was used to carry out the image analysis of the gels.

For the Western blot analysis, unstained SDS-PAGE gels were treated as described by Rutigliano et al. [22], and the SeeBlue™ pre-stained marker (Thermo Fisher Scientific, Waltham, MA, USA) was used as the immunoblotting marker. Specific antibodies to peanut (Ara h 1, Ara h 3, and Ara h 2), gluten (mAb R5, mAb G12, and mAb 0610), and egg (ovalbumin (polyclonal Ab)) were employed. The anti-rabbit HRP conjugate and the anti-mouse alkaline phosphatase conjugate were used as secondary antibodies for peanut allergens and ovalbumin in the case of the former and gluten proteins in the case of the latter. Peanut and ovalbumin blots were developed for 10 min using Super Signal West Dura substrate solutions diluted 1:1 (*v*/*v*), while the gluten blots were developed for 10 min using a phosphate substrate solution diluted 1:2 (*v*/*v*) in milliQ water. Blot images were acquired (Fujifilm LAS-1000, Fuji, Japan), and selected polypeptides reproducibly resolved as discrete bands were considered for the densitometric analysis using ImageJ software [22].

### 2.4. Chromatographic Protein Separation (SE-HPLC)

The molecular weight protein distribution of the starting raw materials, control muffin, 4% peanut muffin, and 20% peanut muffin was assessed by the application of the two-step extraction procedure described by Gupta et al. [23] and Rutigliano et al. [22], testing the efficiency of two extraction buffers: the one described by the original method (Buffer A) [23] was compared with a second buffer (Buffer B), which was obtained considering the addition of denaturing (2 mol L^−1^ Urea) and reducing (1.5% (*w*/*v*) Dithiothreitol, DTT) agents [22]. The “SDS-extractable polymeric proteins” (SDS-EPs) and the “SDS-Unextractable polymeric proteins” (SDS-UPs), i.e., proteins extractable with the aid of sonication, were assessed.

Chromatographic separation was performed using a liquid chromatograph Shimadzu HPLC (LC-20AP) equipped with a Phenomenex Biosep SEC-S4000 column (300 Å~7.8 mm, Phenomenex, Torrence, CA, USA), which was calibrated using protein standards as follows: Cytochrome c (12.4), Carbonic Anhydrase (29.0), albumin, bovine serum (66.0), alcohol dehydrogenase (150.0) and β-Amylase (200) (Sigma-Aldrich, Saint Louis, MI, USA) (see Supplementary Figure S1). All analyses were performed in triplicate (n = 3), and the coefficient of variation (CV%) was calculated as an index of chromatographic reproducibility, which always showed values lower than 10% (min 0.06–max 2.8).

The percentage of total (%tUPPs) and large (%lUPPs) unextractable polymeric proteins was calculated as described by Rutigliano et al. [22].

### 2.5. Statistical Analysis

Data were analyzed using XLSTAT 2024.1.0 software (Addinsoft, New York, NY, USA). The influence of the two buffers (Buffer A and Buffer B) on protein extractability was assessed through a one-way ANOVA test. Tukey’s test was carried out to determine significant differences among samples, with the option of homogeneous groups (*p* < 0.05). Data were presented as the mean of three replicates ± standard deviation (SD).

## 3. Results

### 3.1. Electrophoresis and Western Blot of Raw Matter and Muffin Samples

**Figure 1:**
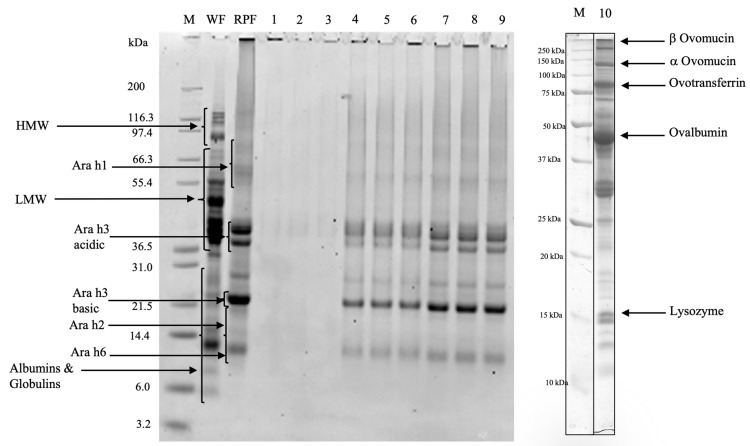
Electrophoretic separation of wheat flour, roasted peanut flour, and muffin total proteins. M: protein marker; WF: wheat flour; RPF: roasted peanut flour; Profiles (1-2-3): control muffins; Profiles (4-5-6): 4% peanut muffins; Profiles (7-8-9): 20% peanut muffins; and Profile 10: egg total proteins.

The results obtained from the electrophoretic separation carried out under reducing conditions of three independent samples of control muffins and 4% and 20% peanut muffins are shown in Figure 1. Most of the samples showed a band that did not enter into the resolving gel, as a result of protein aggregate formation due to baking. According to Rutigliano et al. [22], wheat flour (WF) showed the expected protein electrophoretic separation [24,25]. Moreover, roasted peanut flour (RPF) showed bands related to the main peanut proteins: Ara h3, presenting acid subunits (~40 kDa) and basic subunits (~23 kDa), and Ara h1 (~63 kDa), Ara h2, and Ara h6 (range between 17–14 kDa), with a detected lower intensity [26]. Regarding the egg proteins (Figure 1, Profile 10), the major egg proteins were identified, with yolk proteins including a number of polypeptides spread along the gel on the basis of their molecular weight, ranging from 20 to 200 kDa. They comprised the LDL and HDL apoproteins and other free proteins [27]. In this context, the albumen, with its water content, is more abundant than the yolk, with a ratio yolk/albumen of about 0.47 [28]; therefore, more intense protein bands related to albumen can be shown and identified in the electrophoretic patterns, according to Huopalahti et al. [29] and Guha et al. [30].

**Figure 2:**
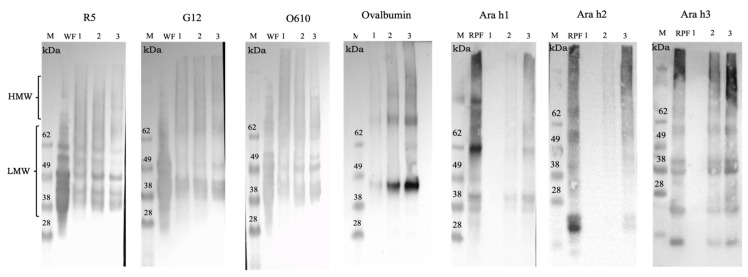
Western blot of muffin samples. M: protein marker; WF: wheat flour; RPF: roasted peanut flour; Profile 1: control muffins; Profile 2: 4% peanut muffins; and Profile 3: 20% peanut muffins analyzed using the R5, G12, and O610 antibodies and against ovalbumin, Ara h1, Ara h2, and Ara h3.

The control muffins (Profiles 1-2-3) showed a hardly detectable protein separation that allowed us to barely detect the wheat, peanut, and egg proteins (Figure 1). In the gluten–water blend with egg whites, heat treatment induced polymerization and losses in extractability greater than those observed for gluten proteins and egg whites analyzed on their own [11]. Therefore, the protein polymerization of the individual components and the interactions among different proteins occurring in the formulation needed to be taken into account. The electrophoretic patterns of the 4% peanut muffins (Profiles 4-5-6) and 20% peanut muffins (Profiles 7-8-9) showed the presence of band proteins from wheat and roasted peanut flours. For these samples, protein bands with a high intensity were detected in the range 38–45 kDa and at 22 kDa, corresponding to the wheat LMW-GS and RPF Ara h3 basic subunit, especially in the case of 20% peanut muffins. The peanut protein bands were much more intense than those related to the wheat flour (Figure 1), which appeared as faint bands. In order to assess the protein immunoreactivity, Western blot detection was performed, and the results are shown in Figure 2. Three antibodies against wheat proteins were used: R5 [31], G12 [32], and IFRN 0610 monoclonal antibody [33].

In this regard, it was possible to highlight that the reactivity decreased as the percentage of roasted peanut flour increased in the samples for all the tested antibodies, as confirmed by the densitometric analysis. Actually, considering the densitometric values of wheat flour (WF) as a reference, an average of percentage decrease of 73.4% for R5, 42.5% for G12, and 26.11% for IFRN O610 was detected when passing from the control muffins (profile 1) to the 20% peanut muffins (profile 3). The occurrence of high-molecular-weight protein aggregates affected the wheat protein solubility and reactivity [22] as a consequence of the interaction between gluten, egg, and peanut proteins. Ovalbumin showed increasing reactivity when passing from the control muffin (Figure 2, Profile 1) to the 20% peanut muffin (Figure 2, Profile 3), indicating that the presence of proteins from different origins in the formulation can lead to different protein–protein interactions and a novel protein architecture. The immunoreactivity of roasted peanut flour allergens (Ara h1, Ara h2, and Ara h3) was assessed by Western blot, considering as the negative sample the control muffin (Figure 2, Profile 1) and peanut flour as the positive sample (Figure 2, Profile 2). As expected, no reactivity was found in the negative sample (i.e., control muffin) in the three immunoblots, while both Ara h1 and Ara h3 (both the acid and basic subunits) were recognized, according to the added percentage of roasted peanut flour. Ara h2 was recognized only in the 20% peanut muffin (Figure 2, Profile 3). From these results, it was possible to highlight how the presence of a protein with a different origin (i.e., eggs) could affect protein matrix organization and interactions among the ingredients, leading to very low protein extractability in the case of the control muffins, although all the most important epitopes had been detected in the blots. On the contrary, the most important peanut proteins started being evident from the electrophoretic separation, increasing their intensity with the increasing percentage of peanut flour in the formulation. These results were also confirmed by the immunoblots, with the exception of Ara h2, whose presence was detected only in the case of 20% peanut muffins. A preliminary hypothesis could highlight the crucial interaction between cereal and egg proteins that creates a “core”, the main protein structure, where peanut proteins can bind.

### 3.2. Study on Protein Extractability Through a Chromatographic Approach

**Figure 3:**
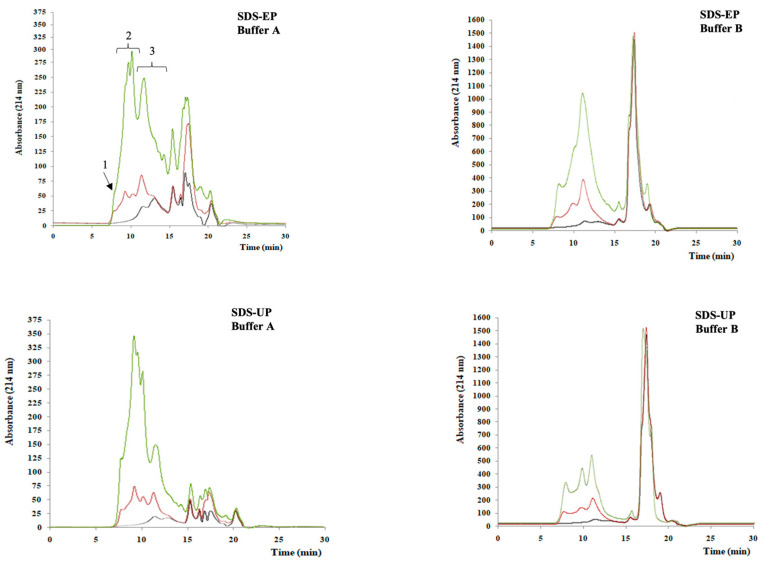
Chromatograms of SDS-EP and SDS-UP of muffin samples with Buffer A and Buffer B. Black lines refer to control muffins, red lines refer to 4% peanut muffins, and green lines refer to 20% peanut muffins.

Protein separation on the basis of molecular weight distribution was accomplished through size-exclusion high-performance liquid chromatography. In this experimentation, two different buffers (Buffer A and Buffer B) were used to further investigate protein aggregate formation in such a complex food matrix, providing new information on protein extractability in cereal-based baked products. SDS-extractable (SDS-EP) and SDS-unextractable (SDS-UP) protein fractions were evaluated, considering either Buffer A or Buffer B (Figure 3). The chromatographic profiles showed the highest absorbance values with the use of Buffer B, with about 1100 mAu vs. about 300 mAu for SDS-EP and about 550 vs. about 350 mAu for SDS-UP. The recognized fractionation of wheat proteins was organized according to the continuous molecular weight distribution: peak 1, between 660 ÷ 200 kDa (large polymeric proteins, LPPs); peak 2, between 200 ÷ 55 kDa (small polymeric proteins, SPPs); peak 3, between 55–30 kDa (large monomeric proteins, LMPs); and peak 4, less than 30 kDa (small monomeric proteins, SMPs) [34,35]. The presence of roasted peanut flour and egg proteins in the batter made the chromatograms more complex than wheat protein alone [22,23,24,25,26,27,28,29,30,31,32,33,34,35,36]. Therefore, the chromatographic profiles shown in Figure 3 should be evaluated considering the following:

- interacting peaks due to the interaction between the sample extraction buffer and the stationary phase elute after 15 min [37,38];
- the not well-resolved peaks obtained with the use of Buffer A were grouped on the basis of the retention time and, therefore, on the basis of their proximity to the estimated molecular weight.

On these bases, three peaks were detected in the chromatographic separation using both Buffers A and B: peak 1, LPPs in the retention time range (RT) of 7.5–9.5 min; peak 2, SPPs in the RT range of 9.5–12 min; and peaks 3 + 4, LMPs and SMPs eluted in the RT range of 12–15 min (Figure 3). It was useful to remember that the SDS-extractable protein (SDS-EP) fraction was dissolved only as a consequence of the buffer’s properties, while the SDS-unextractable protein (SDS-UP) fraction was extracted with the aid of sonication.

**Figure 4:**
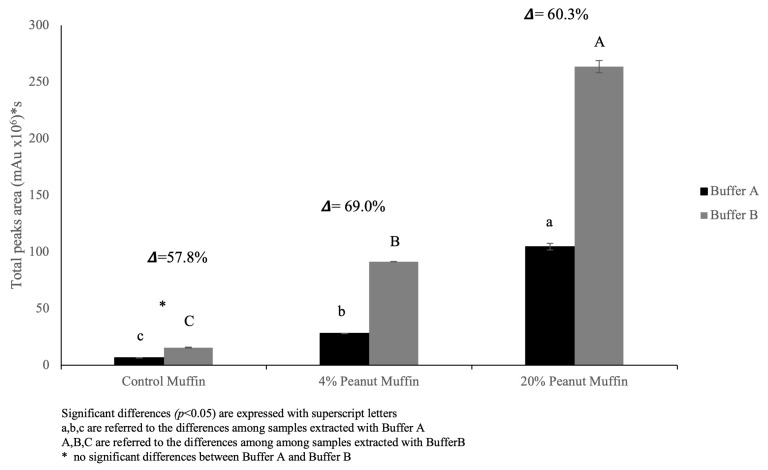
Sum of peak areas considering both the SDS-extractable fraction (SDS-EP) and SDS-unextractable fraction (SDS-UP) calculated for the two tested buffers.

The use of sonication should lead to the disaggregation of the largest insoluble aggregates, increasing their dissolution and, hence, improving the extraction conditions. Generally, the first two peaks represented by polymeric proteins are mainly affected by this occurrence, since they are rich in intermolecular and reducible covalent bonds, which can affect extractability. Considering the architecture of the gluten network developed through these types of linkages [39], the use of urea and DTT (Buffer B) weakened this supramolecular structure in our study, enhancing protein extractability. Moreover, the occurrence of other types of covalent bonds could not be ignored [40]. The inclusion of egg proteins led to a further reduction in protein extractability, reinforcing the protein structure with covalent bonds, in particular with new disulfide and non-disulfide covalent bonds [41,42]. Therefore, the chromatographic separation became more complex because of the increase in protein molecular weight distribution due to the formation of protein aggregates (Figure 3). Protein extractability varied with the employed buffer, and, to better assess this feature, the total area (obtained from the sum of the three detected peaks for the SDS-EP and SDS-UP fractions) was calculated. The different values of protein solubility were expressed as a percentage difference and are illustrated in Figure 4. As expected, the addition of urea and DTT (buffer B) led to a significant increase (*p* < 0.05) in protein extractability in all the samples, although this increase was not revealed for the control muffins’ protein chromatographic separation (Figure 3) and extractability (Figure 4).

**Figure 5:**
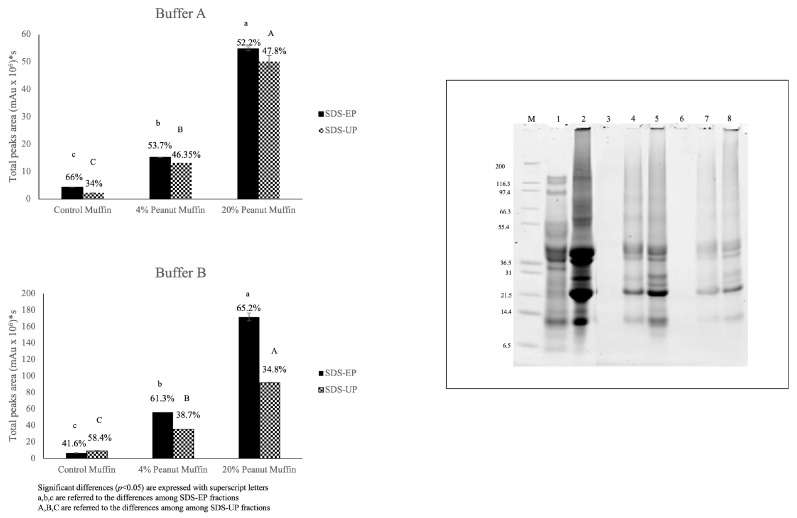
Sum of peak area of SDS-EP and SDS-UP portions extracted with Buffer A and Buffer B. The SDS-PAGE profiles of the SDS-EP and SDS-UP extracted with Buffer B are shown in the box. M: Mark12; 1: wheat flour SDS-EP; 2: roasted peanut flour SDS-EP; 3: control muffin SDS-EP; 4: 4% peanut muffin SDS-EP; 5: 20% peanut muffin SDS-EP; 6: control muffin SDS-UP; 7: 4% peanut muffin SDS-UP; and 8: 20% peanut muffin SDS-UP.

The percentage difference (Δ) calculated between the two buffers was lower in the control muffin (Δ = 57.8%) than in the other samples, namely Δ = 69.0% for the 4% peanut muffin and Δ = 60.3% for the 20% peanut muffin. These results were consistent with those obtained from the electrophoretic analysis (Figure 1), where the protein patterns were revealed especially in the samples employing roasted peanut flour, while they were barely visible for the control sample due to a very low extraction efficiency, probably because of the high rate of aggregation caused by the presence of eggs in the formulation. On this matter, it was noteworthy that both the electrophoretic patterns and the chromatographic profiles of the control muffins suggested the possible formation of a complex food structure, characterized by the presence of sulfur-containing amino acids and egg protein coagulation, which may have resulted in a negligible extraction capacity for the whole protein pattern. The different percentages of extraction of the SDS-extractable (SDS-EP) and SDS-unextractable (SDS-UP) portions are reported in Figure 5, comparing Buffer A and Buffer B. Once again, it is possible to underline how the total peak area detected for the samples extracted with Buffer B was higher (200 × 10^6^ mAu * s) than with Buffer A (60 × 10^6^ mAu * s), indicating the higher extraction efficiency of the former, especially for the 20% peanut muffin. Regarding the control muffin, it showed the lowest extractability with respect to the other samples, with no significant differences in the extraction efficiency between the two buffers, unlike for the proportion of SDS-EP and SDS-UP fractions. A higher percentage of extracted SDS-EP (66%) than SDS-UP (34%) was found with Buffer A, while the opposite was found when Buffer B was used (i.e., 41.6% for SDS-EP and 58.4% for SDS-UP), with a higher extraction efficiency with the aid of urea and DTT, supported by sonication. For the samples comprising 4% and 20% peanut flour, instead, the percentage of the proteins extracted in the SDS-EP fraction was always higher than in the SDS-UP fraction, no matter which buffer was used, but with a completely and statistically different (*p* < 0.05) rate of extraction in the two fractions when comparing the two buffers. These results confirmed the existence of a strong network made of gluten and egg proteins, suggesting that hydrophobic interactions alongside hydrogen and reducing covalent bonds were involved in this architecture. The inclusion of roasted peanut flour in the muffin formulation, with the two different percentages, increased the protein extractability for the two buffers, suggesting the weakening of the gluten network with a decreasing proportion of high-molecular-weight polymeric proteins or by allowing the arrangement of a network covered by peanut proteins, more easily extractable with the employed buffers (Figure 1 and Figure 3). The protein extracts analyzed through SE-HPLC were also analyzed through SDS-PAGE to further validate these hypotheses. The electrophoretic separation of the extracts obtained with Buffer B (i.e., with the best conditions for extraction), for both the SDS-EP and SDS-UP portions, is shown in the box in Figure 5, where, noticeably, no protein bands can be detected in the control muffins (Profile 3 and Profile 6, respectively), although peaks can be detected in the chromatograms.

This was due to the cut-off of electrophoretic separation, which was lower than that allowed by gel filtration chromatography, confirming the presence of large and unextractable protein aggregates, generated by the interaction between wheat proteins and eggs, as a result of baking temperatures. Moreover, the possibility that the electrophoretic technique and/or the gel coloring system was not sensitive enough to detect the presence of protein bands should be taken into account, considering that the detection of protein peaks was very low, alongside that of chromatographic separation. In reality, the electrophoretic pattern of the wheat proteins (Profile 1) was clear, and well-resolved bands related to the main protein fractions (HMW-GS, LMW-GS, gliadins, albumins, and globulins) [24] were detected. The profile related to roasted peanut flour (Profile 2) was quite similar to that shown in the electrophoretic pattern of the total proteins (Figure 1, RPF), although, in this case, the protein bands were more intense and had a better resolution. The electrophoretic patterns of the SDS-EP fractions of the 4% peanut muffin (Profile 4) and 20% peanut muffin (Profile 5) revealed the main bands of roasted peanut flour, with an intensity proportional to the level of fortification, while the protein bands related to wheat flour were barely visible. As expected, the intensity of the protein bands decreased in the SDS-UP fraction, confirming the chromatographic findings.

**Figure 6:**
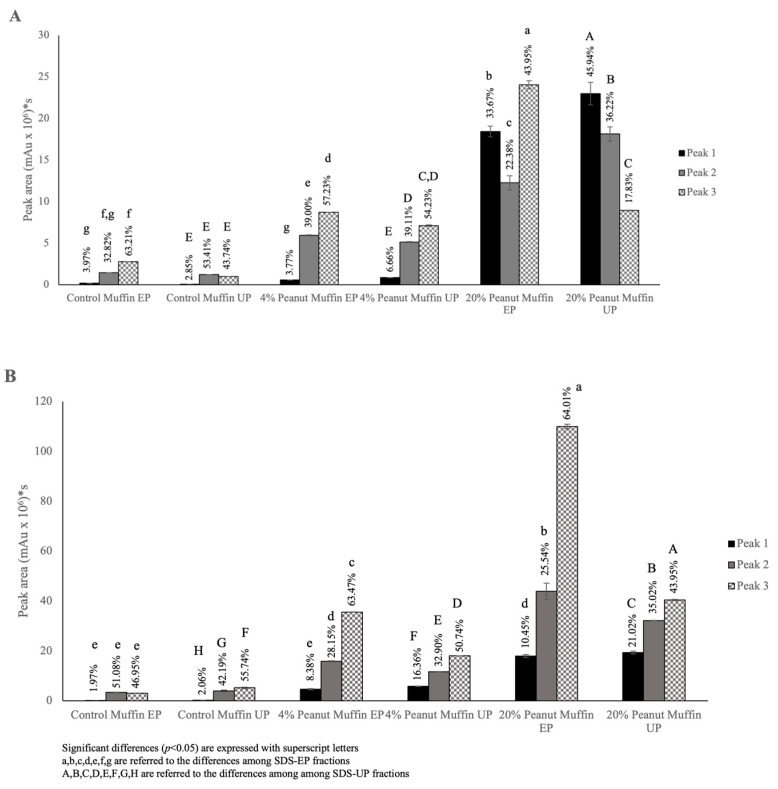
Detail of peak area with relative percentages extracted with Buffer A and Buffer B for the SDS-EP and SDS-UP of the muffin samples.

To further investigate the proteins’ molecular weight distribution, we additionally calculated the percentage of each detected peak for the SDS-EP and SDS-UP fractions in the total extracted area for the two tested buffers (Figure 6A,B), referring to the LPP, SPP, and LMP + SMP peaks as peaks 1, 2, and 3, respectively.

Regarding the extraction carried out using Buffer A (Figure 6A), the control muffins showed a very low percentage related to large polymeric proteins (LPPs, peak 1) for both the SDS-EP and SDS-UP portions, while the highest percentages were registered for peak 3 (LMP + SMP), with 63.21% for SDS-EP, and peak 2 (SPP), with 53.41% for SDS-UP. The muffin samples with the inclusion of 4% roasted peanut flour showed a similar proportion in the protein distribution considering the two fractions (i.e., SDS-EP and SDS-UP), with the highest percentage detected for peak 3 (LMP + SPP), measuring 57.23% and 54.23%, respectively. Moreover, an increase in the LPP peak percentage (peak 1, 6.66%) was highlighted in the SDS-UP fraction. Muffin samples with the inclusion of 20% roasted peanut flour showed the highest percentages for peak 3 (LMP + SMP, 43.95%) in the SDS-EP fraction and peak 1 (LPP, 45.94%) for the SDS-UP fraction, highlighting a marked increase in the extractable polymeric protein (LPP and SPP) forms, especially with the aid of sonication. Three peaks were also recorded using Buffer B, with a protein distribution pushed towards monomeric forms as a consequence of the presence of chaotropic and reducing agents, which contributed to the dissolution of more complex protein structures, releasing lower-molecular-weight proteins (Figure 6, Panel B). A similar protein distribution was registered for the control muffins, which, once again, showed the lowest percentages of large polymeric proteins (LPPs, peak 1). Regarding the 4% peanut muffins, a higher percentage of LPPs was detected than that found in the case of Buffer A (Figure 6, Panel A). A different protein distribution was found for the 20% peanut muffins, where the highest extractability had already been reached in the SDS-EP fraction. This feature suggested the prominence of hydrophobic and covalent reducible linkages and confirmed the extraction of polymeric proteins (LPPs and SPPs), especially with the aid of sonication, since these two percentages were higher in the SDS-UP fraction than the SDS-EP fraction (i.e., 21.02% and 35.02% vs. 10.45% and 25.54%, respectively). Comparing the two buffers, it was found that Buffer B resulted in a significant increase in extractability in the SDS-EP fraction, especially in the case of the peanut muffin samples, while it did not have the same result in the control muffin samples, confirming the results previously shown. For the SDS-UP fraction, however, Buffer B resulted in a significant (*p* < 0.05) increase in extractability in all the tested samples (with the exception of the control muffin, peak 1), probably as a result of the use of sonication in the extraction procedures. Furthermore, the explanation of the different protein distributions could lie in the proteins’ hierarchical supramolecular architecture that, once broken, generates a form of protein distribution with different proportions. Peanut flour impacted protein network arrangement in a different way, based on the added percentage, achieving the extraction of polymeric proteins with both buffers, but this was strengthened by the combination of the urea and DTT buffer with sonication. The similar behavior obtained for the control muffins and 4% peanut muffins was related to the type of batter obtained, where the eggs’ binding capacities played a major role, affecting the extractability of polymeric proteins, considering that these were then also affected by baking [43]. On the other hand, in the case of the 20% peanut muffins, a completely different structure was found, potentially creating an unextractable nucleus, surrounded by peanut proteins, linked through hydrophobic, disulfide, but also non-reducible bonds, as a result of baking [44,45].

**Figure 7:**
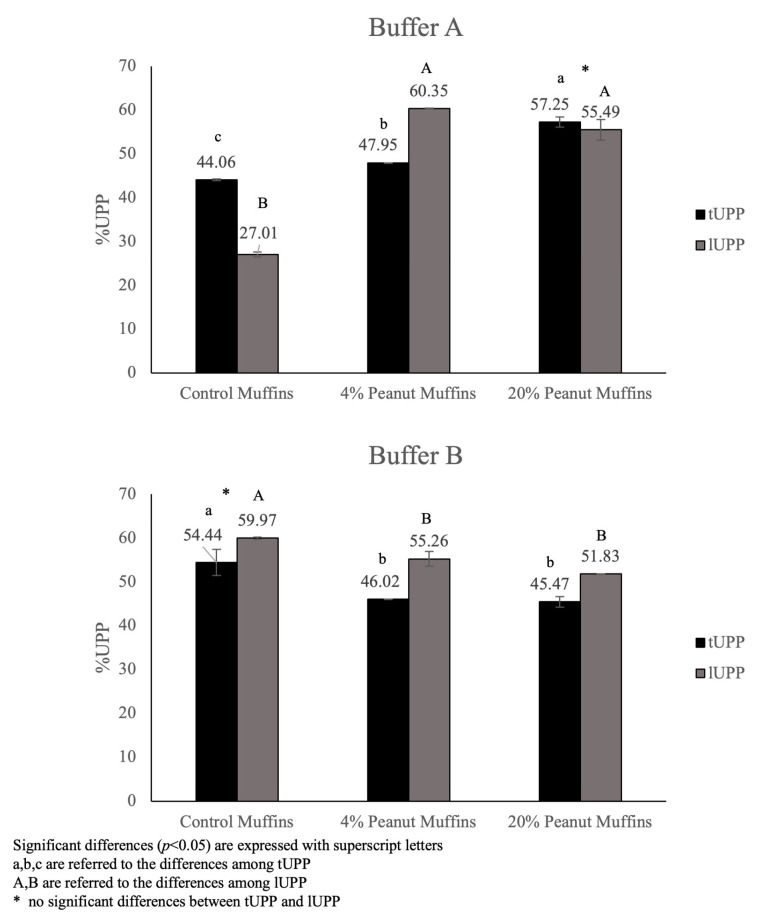
Total (tUPP) and large unextractable polymeric protein (lUPP) percentages of muffin samples obtained with Buffer A and Buffer B.

Finally, the percentages of total and large unextractable polymeric proteins were calculated, because these could be considered important indexes to evaluate the rate of protein aggregate formation [22], and the results are shown in Figure 7. The total unextractable polymeric protein percentage includes the peaks related to the polymeric proteins (LPPs and SPPs), showing the incidence of the unextractable polymeric proteins on the total extracted polymeric proteins, while the second index indicates the formation of large polymeric protein aggregates (LPPs, peak 1) among the total extracted large polymeric proteins (peak 1 of SDS-EP + peak 1 of SDS-UP). The chemical features of Buffer A allowed it to affect hydrophobic interactions, leading to a significant (*p* < 0.05) increase in tUPP and lUPP for the samples prepared with roasted peanut flour. In particular, the percentage of lUPP was higher than tUPP for the 4% peanut muffins, whereas no significant differences were found between tUPP and lUPP in the case of the 20% peanut muffins. Therefore, the network between egg and wheat proteins can be considered to represent the unextractable “core” of a supramolecular network, with peanut proteins bound by hydrophobic interactions, involving large and small polymeric proteins.

The employment of Buffer B revealed a higher percentage of lUPPs than tUPPs for all the samples, highlighting the higher contribution of large polymeric proteins in the aggregate formation. The control muffins showed higher percentages, but not a significant difference between the tUPPs and the lUPPs with Buffer B. The increase in tUPPs and lUPPs suggested that polymeric protein aggregate formation was due to reducible covalent bonds, although the formation of other types of covalent non-reducible links could not be excluded. In actuality, beyond the important role of hydrophobic interactions and the formation of hydrogen bonds, the covalent reducing bond (-S-S) also represented an important crosslink between gluten and egg proteins [36,37,38,39,40,41,42,43,44,45,46], especially considering that ovalbumin is characterized by a high amount of free SH groups [11,12,13,14,15,16,17,18,19,20,21,22,23,24,25,26,27,28,29,30,31,32,33,34,35,36]. Moreover, non-reducible bonds that take place in food, such as iso-peptide bonds and crosslinks originating from dehydro-proteins, tyrosine, or Maillard reactions during heating, must also be considered [47]. In this case, the increase in roasted peanut flour percentage determined a remarkable decrease in the two indexes, with no significant differences between the 4% and 20% peanut muffin samples. Therefore, these findings suggest that the amount of gluten and non-gluten proteins is an important factor that can influence the formation of aggregates, whose extractability can be enhanced with the aid of urea and DTT, combined with sonication. Overall, the results confirmed the hypothesis already suggested on this topic by Rutigliano et al. [22] that the proportion, nature, and way in which wheat proteins can interact with other ingredients, together with the baking conditions, influence food matrix organization.

## 4. Discussion

An English muffin recipe, including eggs and roasted peanut flour, was used in this experiment to evaluate the changes occurring in protein organization when three main protein classes interact: wheat, peanut, and egg proteins. In such gluten-based batters, different protein types coexist, and, thus, the manufacturing process can influence their interactions and solubility, determining, consequently, their assembly in supramolecular structures, which is enhanced by the production technology (i.e., baking) [48,49]. In this regard, Rutigliano et al. [22], through an SE-HPLC study of biscuits (a dried baked product), observed reduced extractability due to protein aggregation and found that the addition of other ingredients (i.e., roasted peanut flour) could modify the protein network. In this study, the initial formulation used for a dried baked product [22] was further enriched with eggs to produce a wet cereal-based baked product (i.e., muffins). SDS-PAGE showed the highest protein extractability for roasted peanut flour proteins in both formulations (4% and 20%), while wheat and egg proteins were barely detected in the electrophoretic patterns. These outcomes allowed us to hypothesize a form of protein organization characterized by a strong gluten–egg protein “core” that included and was surrounded by peanut proteins, which were linked through the covalent bonds generated by mixing and baking [44,45,46], but also involved non-covalent linkages.

The evaluation of how immunoreactivity could change in this type of wet cereal-based baked product showed that the intensity of gluten protein epitopes decreased from the control to the 20% peanut muffins, because the amount wheat flour was lower, and following baking. Ovalbumin was weakly detected in the control muffins [50], verifying the strong network established between gluten and egg proteins, although its intensity increased with the increasing amount of roasted peanut flour. On this matter, Deleu et al. [36,37,38,39,40,41,42,43,44,45,46] established that a protein network based on both disulfide bonds and hydrophobic interactions is formed during the baking of pound cakes and that this covalent network includes almost all egg white proteins and most of the yolk and wheat flour proteins.

The presence of roasted peanut flour led to the formation of high-molecular-weight polymeric proteins, since bands were detected especially in the blot related to Ara h3 in the peanut-enriched muffins. Peanut allergens were recognized in both of the tested formulations, with the 20% peanut muffin showing the highest band intensity for all the tested allergens. However, it should be noted that the reactivity of Ara h1 was similar to that of Ara h2, although the former is characterized by one cysteine residue [51] while the latter is characterized by eight cysteine residues [52], and the highest reactivity was observed for Ara h3, characterized by two cysteine residues [53]. Therefore, a deeper study of how the protein architecture could influence the immunoreactivity of the main allergens in this type of product should be undertaken.

Different protein interactions and, therefore, different protein aggregation machineries in these wet cereal-based baked products were determined by the chromatographic evaluation of protein extractability by testing two different extraction buffers. Baking [43] and gluten protein network organization [54], which can be influenced by new ingredients in cereal-based products [55,56], influenced protein extractability in the samples.

The use of two different buffers allowed us to highlight how the inclusion of a strong reducing agent (i.e., DTT) determined an increase in protein extractability, especially in the peanut-enriched muffin samples, but not in the control muffins, confirming the findings of Schirmer and Scherf [44]. A change in the proportion between SDS-extractable and SDS-unextractable proteins was found in the control samples extracted with Buffer B, with more proteins extracted only after sonication. The great increase in polymeric proteins (LPPs and SPPs) in the 4% and 20% peanut muffins just from using Buffer A denoted a notable presence of hydrophobic interactions, whereas the use of Buffer B led to a simplification of the aggregate forms, leading to an increase in the detected monomeric forms, highlighting the occurrence of covalent reducible and hydrogen bonds. The rate of protein aggregation, which was evaluated through the calculation of total and large unextractable polymeric proteins extracted with the two tested buffers, highlighted a higher presence of high-molecular-weight protein aggregates characterized by disulfide bonds and hydrophobic interactions in the control muffins, but it did not greatly modify the proportion of tUPPs and lUPPs in the peanut-enriched muffin samples, where a protein architecture surrounded by peanut proteins was assumed. To the best of our knowledge, no studies about this calculation on muffin samples had been carried out prior to our experiment; therefore, these results are hereby suggested to be the first findings on this topic.

## 5. Conclusions

A possible protein network organization in muffin samples, characterized by a protein blend and baking, was assumed from the results obtained from our study of protein extractability. By testing two different buffers, the highest rate of extraction was obtained when denaturing (urea) and reducing (DTT) agents were employed, although the control muffins showed the lowest protein extractability, no matter which working buffer was used. Therefore, a further investigation to obtain the best extraction conditions for cereal-based baked products is ongoing.

Our analysis of the blots suggested that Ara h1 and Ara h2 could take part in intermolecular aggregation events, affecting the response against antibodies and, therefore, protein immunoreactivity, while the immunoreactivity of Ara h3 was less influenced. The increased intensity of ovalbumin in the blots, when the highest percentage of roasted peanut flour was used, confirmed that the presence of this flour could interfere with the gluten–egg “core”. Thus, a central “core” comprising gluten–egg protein aggregates surrounded by peanut proteins through hydrophobic interactions and disulfide bridges, as well as hydrogen bonds, was suggested.

Further information on protein allergens’ engagement in supramolecular structures and their delivering properties in wet cereal-based baked products could be assessed by evaluating the composition of polymeric aggregates through mass spectrometry and in vitro digestion tests.

## Figures and Tables

**Table 1:** Muffin formulation.

Ingredients	Control Muffins (g)	4% Peanut Muffins (g)	20% Peanut Muffins (g)
Soft Wheat Flour	400 (40%)	360 (36%)	200 (20%)
Roasted Peanut Flour	0	40 (4%)	200 (20%)
Soft Caster Sugar	200 (20%)	200 (20%)	150 (15%)
Vegetable Oil	90 (9%)	90 (9%)	90 (9%)
Medium Eggs	100 (10%)	100 (10%)	100 (10%)
Sodium Bicarbonate	6.5 (0.65%)	6.5 (0.65%)	6.5 (0.65%)
Vanilla Essence	0.5 (0.05%)	0.5 (0.05%)	0.5 (0.05%)
Salt	3 (0.3%)	3 (0.3%)	3 (0.3%)
Warm Water	200 (20%)	200 (20%)	250 (25%)
Total	1000 (100%)	1000 (100%)	1000 (100%)

## Data Availability

The original contributions presented in this study are included in the article; further inquiries can be directed to the corresponding author.

## Supplementary Materials

The following supporting information can be downloaded at https://www.mdpi.com/article/10.3390/foods14040710/s1: Figure S1: SE-HPLC standard elution; Table S1: Protein content of muffin samples.

## Abbreviations

The following abbreviations are used in this manuscript:

WF: wheat flour; RPF: roasted peanut flour; LPPs: large polymeric proteins; SPPs: small polymeric proteins; LMPs: large monomeric proteins; SMPs: small monomeric proteins; SDS-EP: SDS-extractable fraction; SDS-UP: SDS-unextractable fraction; tUPPs: total unextractable polymeric proteins; and lUPPs: large unextractable polymeric proteins
