# Supplementary material for: Influence of Peanut Flour Enrichment and Eggs on Muffin Protein Aggregation
Source: Foods. 2025 Feb 19;14(4):710. doi: 10.3390/foods14040710 (PMC11854293; doi:10.3390/foods14040710)
Supplement: Supplementary file 1 [file foods-14-00710-s001.zip › Supplementary Materials/Supplementary Table 1.docx]

**Table S1-** Protein content of muffin samples

| Samples | % crude protein |
| --- | --- |
| Control Muffin | 4.89±0.57^c^ |
| 4% Peanut Muffin | 7.11±0.05^b^ |
| 20% Peanut Muffin | 14.06±0.30^a^ |

a,b,c *p*<0.05
