# Supplementary figures and images for: Influence of Peanut Flour Enrichment and Eggs on Muffin Protein Aggregation
Source: Foods. 2025 Feb 19;14(4):710. doi: 10.3390/foods14040710 (PMC11854293; doi:10.3390/foods14040710)

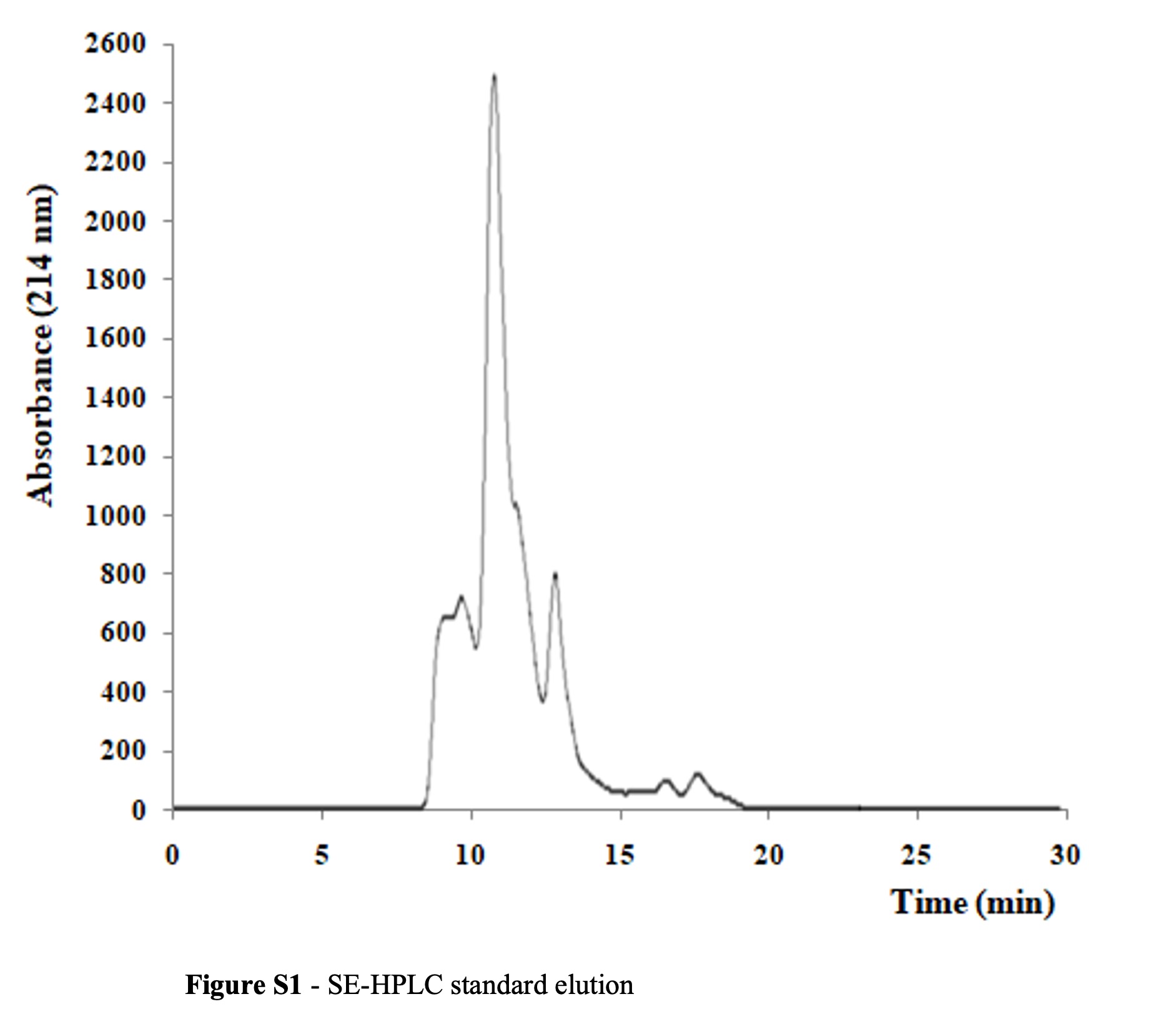

Supplement: Supplementary file 1 [file foods-14-00710-s001.zip › Supplementary Materials/FigureS1.jpg]
